# Modification of picornavirus genomic RNA using ‘click’ chemistry shows that unlinking of the VPg peptide is dispensable for translation and replication of the incoming viral RNA

**DOI:** 10.1093/nar/gkt1162

**Published:** 2013-11-15

**Authors:** Martijn A. Langereis, Qian Feng, Frank H. T. Nelissen, Richard Virgen-Slane, Gerbrand J. van der Heden van Noort, Sonia Maciejewski, Dmitri V. Filippov, Bert L. Semler, Floris L. van Delft, Frank J.M. van Kuppeveld

**Affiliations:** ^1^Virology Division, Department of Infectious Diseases and Immunology, Faculty of Veterinary Medicine, Utrecht University, Utrecht, 3584 CL, The Netherlands, ^2^Institute for Molecules and Materials, Radboud University Nijmegen, Nijmegen, 6500 HB, The Netherlands, ^3^Department of Microbiology and Molecular Genetics, School of Medicine, University of California, Irvine, CA 92697, USA and ^4^Leiden Institute of Chemistry, Leiden University, Bioorganic Synthesis Leiden, 2300 RA, The Netherlands

## Abstract

Picornaviruses constitute a large group of viruses comprising medically and economically important pathogens such as poliovirus, coxsackievirus, rhinovirus, enterovirus 71 and foot-and-mouth disease virus. A unique characteristic of these viruses is the use of a viral peptide (VPg) as primer for viral RNA synthesis. As a consequence, all newly formed viral RNA molecules possess a covalently linked VPg peptide. It is known that VPg is enzymatically released from the incoming viral RNA by a host protein, called TDP2, but it is still unclear whether the release of VPg is necessary to initiate RNA translation. To study the possible requirement of VPg release for RNA translation, we developed a novel method to modify the genomic viral RNA with VPg linked via a ‘non-cleavable’ bond. We coupled an azide-modified VPg peptide to an RNA primer harboring a cyclooctyne [bicyclo[6.1.0]nonyne (BCN)] by a copper-free ‘click’ reaction, leading to a VPg-triazole-RNA construct that was ‘non-cleavable’ by TDP2. We successfully ligated the VPg-RNA complex to the viral genomic RNA, directed by base pairing. We show that the lack of VPg unlinkase does not influence RNA translation or replication. Thus, the release of the VPg from the incoming viral RNA is not a prerequisite for RNA translation or replication.

## INTRODUCTION

Picornaviruses constitute a large group of small non-enveloped RNA viruses including many important human and animal pathogens like poliovirus (PV), coxsackievirus, rhinovirus, enterovirus 71 and foot-and-mouth disease virus. These viruses possess a single-stranded RNA genome of positive polarity ranging from 7.0 to 8.5 kb in length. The genome consists of a single open reading frame flanked by two highly structured untranslated regions (UTRs). The 5′ UTR contains the internal ribosomal entry site essential for viral RNA translation (1–3). Additionally, at the ultimate 5′ terminus another element—the cloverleaf (CL) structure—is present, which is involved in both viral RNA translation and replication (4–8).

A unique feature of picornaviruses is the presence of a small virally encoded peptide, VPg (also known as 3B), at the 5′ terminus of the genomic RNA (9–11). This peptide plays a key role in the RNA replication process. Once VPg is released from the viral polyprotein by the 3C^pro^ proteinase (12), the tyrosine residue at position 3 of VPg is uridylylated by the 3D viral polymerase (13,14). The resulting VPg-pUpU then serves as a primer for both negative-sense as well as positive-sense RNA transcription (15). As a consequence, all newly transcribed RNA copies contain VPg attached via a covalent phosphodiester bond (9–11).

Soon after the discovery that picornavirus virion RNA contained covalently linked VPg, viral RNA molecules lacking VPg were detected in infected cells (15–17). Thus, VPg seems to be released from the virion RNA early in the viral replication cycle. This so-called ‘unlinkase’ activity was also identified in lysates from uninfected cells (18–20), suggesting that a host enzyme is responsible for the unlinkase event. Recently, the 5′-tyrosyl-DNA phosphodiesterase-2 (TDP2) enzyme was identified as the long-sought ‘unlinkase’ enzyme (21).

The function of VPg release from the genomic viral RNA, if any, is still unknown. Clearly, VPg is not essential for the translation of incoming viral RNA because proteinase K treatment of genomic viral RNA, thereby truncating the VPg peptide, did not affect translation efficiency (16,22). Moreover, *in vitro* transcribed RNA, consequently lacking VPg, is translated and replicated efficiently. Because VPg is released from the viral RNA on introduction in the cytoplasm, it has been suggested that the presence of the VPg may hamper the formation of the translation initiation complex (15,16,23). In line with this hypothesis, analysis of viral RNA molecules that associated with ribosomes in infected cells lacked VPg (16,17), although in another study, it was demonstrated that VPg-containing RNA was able to form complexes with ribosomes (24). Therefore, it remains unclear whether the release of VPg from the viral genomic RNA is a prerequisite for translation and/or RNA replication.

It is technically challenging to modify the 5′ terminus of large RNA molecules. Here, we describe a new methodology to decorate the 5′ terminus of the genomic viral RNA with several different modifications. Using this methodology, we show that small modifications at the 5′ terminus do not affect RNA translation and replication. Moreover, we generated viral RNA containing VPg linked via a ‘non-cleavable’ bond. We show that this RNA is efficiently translated and replicated, suggesting that VPg unlinkase is not required for these processes.

## MATERIALS AND METHODS

### Cells and infectious clones

HeLa R19 cells were maintained in Dulbecco’s modified Eagle’s medium supplemented with 10% fetal calf serum, penicillin (10 U/ml) and streptomycin (10 µg/ml). The coxsackievirus strain B3 (CVB3) infectious clone encoding the *Renilla* luciferase (RLuc) gene under control of the viral internal ribosomal entry site [RLuc-CVB3, (25)] was used as a template for site-directed mutagenesis (Stratagene). First, the coding sequence of the first 6 nt of the CL structure was deleted (forward [Fw]: 5′-TAATACGACTCACTATAGG/CAGCCTGTGGGTTGATC-3′ and reverse [Rv]: 5′-GATCAACCCACAGGCTG/CCTATAGTGAGTCGTATTA-3′). The deletion was confirmed by sequence analysis, and the resulting construct was used for another site-directed mutagenesis to introduce either an 8-nt insertion (underlined) to yield the RLuc-CVB3-Δ1-6 + 8 infectious clone (Fw: 5′-CTTTGTGCGCCTGTTTTAGCGGTGGATACCCCCTCCCCCA-3′ and Rv: 5′-TGGGGGAGGGGGTATCCACCGCTAAAACAGGCGCACAAAG-3′) or a 5-nt insertion (underlined) yielding the RLuc-CVB3-Δ1-6 + 5 infectious clone (Fw: 5′-CGGTACCTTTGTGCGCCTGCCCTGTTTTATACCCCCTCCCCCAAC-3′ ;and Rv: 5′-GTTGGGGGAGGGGGTATAAAACAGGGCAGGCGCACAAAGGTACCG-3′). Again, the correct insertion was confirmed by sequencing.

### RNA primers

Unmodified RNA primers, containing a 5′-terminal hydroxyl (OH) group, as well as RNA primers possessing a 5′biotin, amine or Cy5 modification, were purchased from Sigma. The RNA primer modified with bicyclo[6.1.0]nonyne (BCN) (26,27) was synthesized by IBA using a BCN-phosphoramidite building block from B&A. Synthesis of the three RNA primers containing a partial VPg peptide linked via; the natural tyrosine phosphodiester bond—GH[5], H-Gly-Ala-Tyr-Thr-Gly-NH2; GH[9], Ac-Gly-Ala-Tyr-Thr-Gly-NH2; and GH[11], Ac-Ala-Tyr-OH—has been described (28).

### RNA transcription and RNA primer ligation

The 250-nt RNA fragment used for RNA ligations was produced by run-off RNA transcription using the T7 RiboMAX kit (Promega) for 2 h at 37°C. As template, a 250-bp PCR product was used (Fw: 5′-TAATACGACTCACTATAGG-3′ and Rv: 5′-GTAGTTGGCCGGATAACGAACG-3′) spanning the 5′-end of the RLuc-CVB3-CLΔ1-6 + 8 genome containing a T7 promoter sequence. For transcription of genomic RLuc-CVB3 wild-type (wt) RNA, RLuc-CVB3-Δ1-6 + 8 RNA and RLuc-CVB3-Δ1-6 + 5 RNA, the infectious clone was linearized with BamHI, and run-off RNA transcripts were made using the T7 RiboMAX kit (Promega) for 2 h at 37°C. RNA transcripts were purified and concentrated using LiCl precipitation (Ambion) followed by polyphosphatase treatment (Epicentre) for 2 h at 37°C. Finally, the RNA was purified once more using LiCl precipitation (Ambion) and used for RNA ligations.

For RNA primer ligation, 200 pmol of the 12-nt RNA primers (5′-UUCCACCGCUAA-3′) or 9-nt RNA primers (5′-UUAAAACAG-3′) was incubated with 20-pmol genomic RNA in the presence of 20 U of RNA ligase 2 (NEB) for 4 h at 37°C (total volume 50 µl). Excess RNA primer was washed away using a GenElute Mammalian total RNA miniprep column (Sigma). To assess RNA primer ligation efficiency, a 250-nt RNA fragment was released from 1.25 pmol of genomic RNA ligation product using DNA primer-directed RNase H digestion (10-µl volume). RNA was incubated with 12.5 pmol of DNA primer (5′-GTAGTTGGCCGATAACGAACG-3′) and 5 U of RNase H (Fermentas) for 20 min at 50°C. The released RNA fragment was analyzed on an 8-M urea, 8% polyacrylamide gel electrophoresis (PAGE) gel and stained with Stains-All (Sigma).

### RNA translation and replication assay

To determine RLuc translation levels, 100 ng of genomic RLuc-CVB3 run-off transcript RNA (wt) or RNA ligation product were transfected (in triplicate) into 100 000 HeLa cells using Lipofectamine2000 (Invitrogen) in the presence of 2.5 mM GuHCl. Cells were lysed in passive lysis buffer (Promega) 8 h post-transfection, and RLuc values were measured using the *Renilla* luciferase assay system according to manufacturer’s instructions (Promega).

To measure RNA replication, 1 ng of genomic RLuc-CVB3 run-off transcript RNA (wt) or RNA ligation product were transfected (in triplicate) into 100 000 HeLa cells using Lipofectamine2000 (Invitrogen) in the presence or absence of 2.5 mM GuHCl. Cells were lysed in passive lysis buffer (Promega) 8 h post-transfection, and RLuc values were measured using the *Renilla* luciferase assay system (Promega). The increase of RLuc values in the absence of replication inhibitor GuHCl illustrates RNA replication.

### RNA stability assay

To determine RNA stability, 100 ng of genomic RLuc-CVB3 run-off transcript RNA (wt) or RNA ligation product were transfected (in triplicate) into 100 000 HeLa cells using Lipofectamine2000 (Invitrogen) in the presence of 2.5 mM GuHCl. At 1, 4 and 8 h post-transfection, total RNA was isolated using the GenElute mammalian total RNA miniprep kit (Sigma-Aldrich). Isolated RNA was treated with DNAse I (Invitrogen) before reverse transcription. cDNA synthesis was performed with the TaqMan reverse transcription reagents kit (Applied Biosystems) using random hexamer primers. Quantitative analysis of viral RNA levels was performed using the LightCycler 480 (Roche).

### Strain-promoted alkyne–azide cycloaddition reaction

To couple the VPg peptide to the BCN-containing RNA primer, a strain-promoted alkyne–azide cycloaddition (SPAAC) was used. Azide-modified CVB3 VPg (H-Gly-Ala-X-Thr-Gly-Val-Pro-Asn-Gln-Lys-Pro-Arg-Val-Pro-Thr-Leu-Arg-Gln-Ala-Lys-Val-Gln-OH) and poliovirus VPg (PV; H-Gly-Ala-X-Thr-Gly-Leu-Pro-Asn-Lys-Lys-Pro-Asn-Val-Pro-Thr-Ile-Arg-Thr-Ala-Lys-Val-Gln-OH) were synthesized by ALMAC (X = γ-azidohomoalanine residue), and a 100-fold excess of VPg (225 nmol) was incubated with 2.25 nmol of BCN group containing RNA primer (IBA) in a total volume of 50 µl of phosphate-buffered saline for 1 h at room temperature. Coupling efficiency of the peptides to BCN was determined by 8-M urea, 20% PAGE gel analyses and stained with Stains-All (Sigma). VPg-containing RNA primers were used for RNA ligation reactions.

### Dot blot analysis VPg-containing genomic RNA

Equimolar amounts (5, 0.5 and 0.05 pmol) of VPg peptides and VPg-containing RNA ligation products were spotted on an Immobilon-P membrane (Millipore). Membranes were washed once with washing buffer (PBS + 0.1% Tween20) and incubated 1 h in blocking buffer (PBS + 0.1% Tween20 + 5% bovine serum albumin). Membranes were successively incubated for 1 h with a rabbit polyclonal antibody (1:500) raised against the PV VPg (29) followed by 30-min incubation with goat-anti-rabbit-HRPO conjugate (BIO-RAD, 1:10 000) diluted in blocking buffer. In between and after the incubations, the membranes were washed, thrice each time, with washing buffer. Finally, membranes were washed once with PBS, incubated with ECL (Amersham) and scanned using the Odyssey Imager (LI-COR).

### VPg unlinkase assay

The VPg unlinkase reaction was essentially performed as described before (21,30) with slight modifications. As positive control, viral RNA isolated from PV virions was used (wt vRNA, gift from Wilfried Bakker). For the unlinkase reaction, 2 pmol of RNA was incubated in 50 µl of unlinkase buffer (20 mM Tris–HCl, pH 7.5, 2 mM MgCl_2_, 1 mM dithiothreitol, 5% v/v glycerol) in the presence or absence of 1 pmol of GST-TDP2 enzyme. Unlinkase reactions were performed for 30 min at 30°C, and treated RNA was purified over a GenElute Mammalian total RNA miniprep column (Sigma). Purified RNA was used for dot blot analysis, and VPg was detected using an antibody raised against the PV VPg (as described earlier). Signal intensity was quantified using the supplied Image Studio Software of LI-COR.

### RNA translation assay in rabbit reticulocyte lysate and HeLa S10 extracts

Rabbit reticulocyte lysate (RRL) from Promega was used for *in vitro* translation. RRL mixtures (in triplicate) were pre-incubated at 30°C for 5 min, and RNA (1 ng/µl final concentration) was added. Reactions were incubated for 30 min at 30°C, translation was stopped by adding a 10-fold excess of H_2_O, and the RLuc values were measured using the *Renilla* luciferase assay system (Promega).

Translation in HeLa S10 extract was performed as previously described (31–33). RNA (0.1 ng/µl final concentration) was added to HeLa S10 extract in triplicate and incubated 30 min at 34°C. Adding a 10-fold excess of H_2_O stopped the translation reaction, and the RLuc values were measured using the *Renilla* luciferase assay system (Promega).

## RESULTS

### Use of the CL structure allows efficient ligation of 5′-modified RNA primers to RLuc-CVB3 genomic RNA

**Figure 1.**
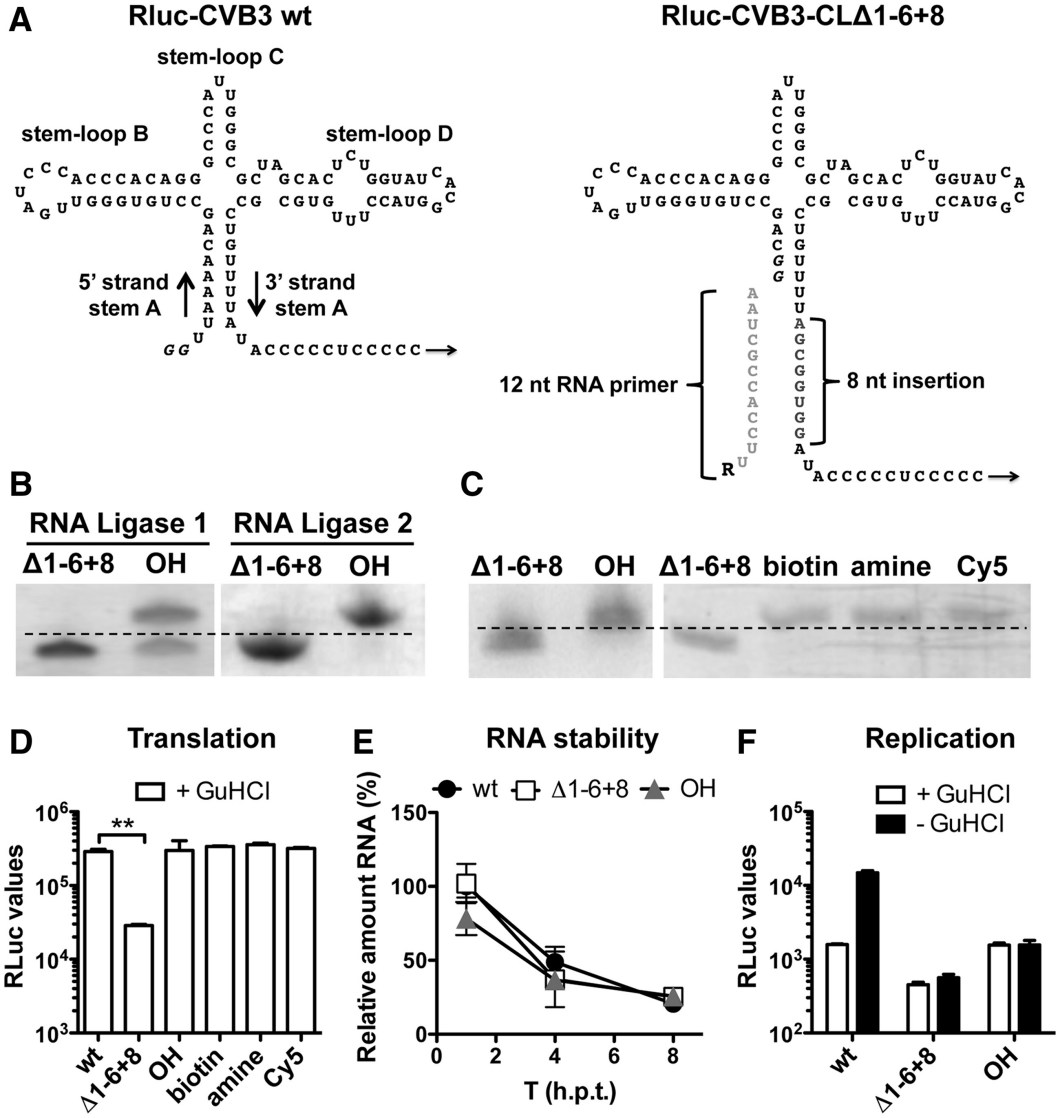
Base pairing-directed RNA primer ligation to RLuc-CVB3-CLΔ1−6 + 8 genomic RNA. (**A**) Schematic representation of the wt and the CLΔ1 − 6 + 8-modified CL structure. The 8-nt insertion in the 3′strand of stem A is indicated in gray. Note that T7 RNA polymerase-transcribed RNA contains two additional guanine nucleotides (*italic*), which form a wobble base pair with uracil nucleotides of the 3′strand of stem A in the Δ1 − 6 + 8-modified CL structure. The 12-nt RNA primer used for base pairing-directed RNA ligation is depicted in light gray, and ‘R’ represents the different 5′ modifications. (**B**) Urea–PAGE analysis of an RNA primer ligation to a 250-nt RNA fragment possessing the Δ1−6 + 8-mutated CL structure. RNA primer was ligated using either RNA Ligase 1 or RNA Ligase 2. Clearly, the RNA Ligase 2 was more efficient in ligating the RNA primer to the modified CLΔ1−6 + 8 structure. (**C**) RNA primer ligation efficiency to genomic RNA possessing the CLΔ1−6 + 8 structure was determined by urea–PAGE analysis of a 250-nt RNase H-digested 5′-terminal fragment. Note that ligation of the RNA primer reduces migration speed of the 250-nt RNase H-digested RNA fragment. (**D**) Translation of the incoming genomic RLuc-CVB3 RNA (wt), RNA holding the mutated CL (Δ1−6 + 8) and RNA ligation products with different 5′ ;modifications (OH, amine, biotin, Cy5) were determined by transfection of RNA in HeLa cells in the presence of GuHCl. Eight hours post-transfection, HeLa cells were lysed and RLuc values were determined. Data from a representative experiment are presented as the mean of duplicate ±SD and analyzed using unpaired *t*-test (** indicates significant difference *P* < 0.01). (**E**) Stability of RLuc-CVB3 RNA (wt), the mutated CL (Δ1−6 + 8) and RNA ligation product possessing a 5′hydroxyl group (OH) was followed over time. RNA was transfected into HeLa cells. At 1, 4 and 8 h post-transfection, cells were lysed and intracellular viral RNA level was analyzed using RT-qPCR. Relative RNA levels are shown in percentages compared with RLuc-CVB3 RNA (wt) at T = 1 h. (**F**) RNA replication of the transfected RNA was determined by comparing the RLuc values in the presence of GuHCl inhibitor (+GuHCl) and in the absence of the inhibitor (-GuHCl). Note that the extended stem of the CLΔ1-6 + 8 structure hampered RNA replication. Data from a representative experiment are presented as the mean of duplicate ±SD.

To investigate the importance of VPg unlinkase for viral RNA translation and replication, we made use of an infectious clone of CVB3 containing *Renilla* luciferase upstream of the capsid region (RLuc-CVB3) (25). Initial attempts to modify the 5′ terminus of the RLuc-CVB3 genomic RNA via ligation of a modified RNA primer were unsuccessful (data not shown). This was most likely the result of low ligation efficiency. To optimize RNA primer ligation, we took advantage of stem A of the CL structure (Figure 1A) for base pairing-directed primer ligation. To this end, we generated an RLuc-CVB3 infectious clone to transcribe RNA containing a mutated CL lacking the first 6 nt in the 5′ strand and containing an insertion of 8 nt in the 3′strand of stem A (CLΔ1-6 + 8, Figure 1A). This RNA should allow annealing of a 12-nt RNA primer with sequence complementary to the 3′strand (Figure 1A).

To validate this base pairing-directed RNA primer ligation procedure, an unmodified 12-nt RNA primer was ligated to a 250-nt RNA fragment harboring the CLΔ1-6 + 8-modified CL structure. For this RNA ligation reaction, two different enzymes were tested, and ligation efficiency was visualized by separating the RNA ligation products by polyacrylamide gel electrophoresis in the presence of urea (urea-PAGE). As shown in Figure 1B, >50% of the 250-nt RNA fragment was modified with the RNA primer using RNA ligase 1. Yet, using RNA ligase 2, which has a preference for dsRNA substrates, allowed ligation efficiency near 100% (Figure 1B). Thus, the annealing of the RNA primer to the CLΔ1-6 + 8 modified CL structure, in combination with the RNA ligase 2 enzyme, significantly increased ligation efficiency as compared with the standard RNA ligation protocol. This new procedure now allows the modification of the RLuc-CVB3 genomic RNA.

To modify the 5′ terminus of the full-length RLuc-CVB3 genomic RNA, several 12-nt RNA primers containing different 5′ termini (R = OH, biotin, amine or Cy5) were ligated to the genomic RLuc-CVB3-CLΔ1-6 + 8 RNA. Because the RLuc-CVB3 genomic RNA is too large (>7500 nt) to allow differentiation of a 12-nt modification on a urea-PAGE gel, a 250-nt RNA fragment was released from the genomic RNA using DNA primer-directed RNase H digestion. Analysis of these 250-nt fragments showed efficient ligation of all different RNA primers to the genomic RLuc-CVB3 genomic RNA (Figure 1C). In conclusion, using the CLΔ1-6 + 8-modified CL structure, we were able to ligate 12-nt RNA primers to the genomic RNA and thereby introducing different modifications to the 5′ terminus.

### Small modifications at the 5′ terminus of genomic RNA do not affect translation

To determine whether the different RNA modifications affect translation, an RLuc-translation assay was performed. To ensure that we only measured translation of the incoming RNA, the RNA ligation products were transfected into HeLa cells in the presence of the replication inhibitor guanidine hydrochloride ( + GuHCl) to prevent RNA replication. Modification of the CL structure (CLΔ1-6 + 8) hampered RLuc translation significantly (Figure 1D). Restoring the CL structure by RNA primer ligation returned RLuc translation to levels similar to those of wt RNA, which suggests that an intact CL stem A is essential for efficient protein translation. The differences in RLuc translation levels were not attributed to changes in RNA stability as result of the mutated CL structure, as intracellular RNA levels were comparable over time (Figure 1E). Importantly, none of the 5′modifications (biotin, amine or Cy5) affected RLuc translation as compared with RNA lacking a 5′modification (OH) or the wt RNA, which possesses two additional guanine nucleotides and a triphosphate 5′ terminus (Figure 1D). Notably, the biotin, amine and Cy5 modifications are not linked via a phosphodiester bond to the RNA and are therefore not released by the TDP2 enzyme. Thus, small modifications at the 5′ terminus of the genomic RNA do not affect translation initiation.

To investigate whether modifications at the 5′ terminus of the genomic RNA influence replication, RNA ligation products were transfected in HeLa cells but this time also in the absence of GuHCl (−GuHCl). This allows translation and also replication of the RNA, which can be measured by an increase in RLuc values. When wt RLuc-CVB3 RNA was transfected in HeLa cells without any drug addition, increased RLuc values were measured (see wt, Figure 1F). Unfortunately, the RNA ligation products that possessed the 8-nt extension of stem A (OH) failed to replicate (Figure 1F), thereby precluding the analysis of 5′-terminal modifications on RNA replication.

### Small modifications at the 5′ terminus of RLuc-CVB3 RNA do not affect translation and replication

**Figure 2.**
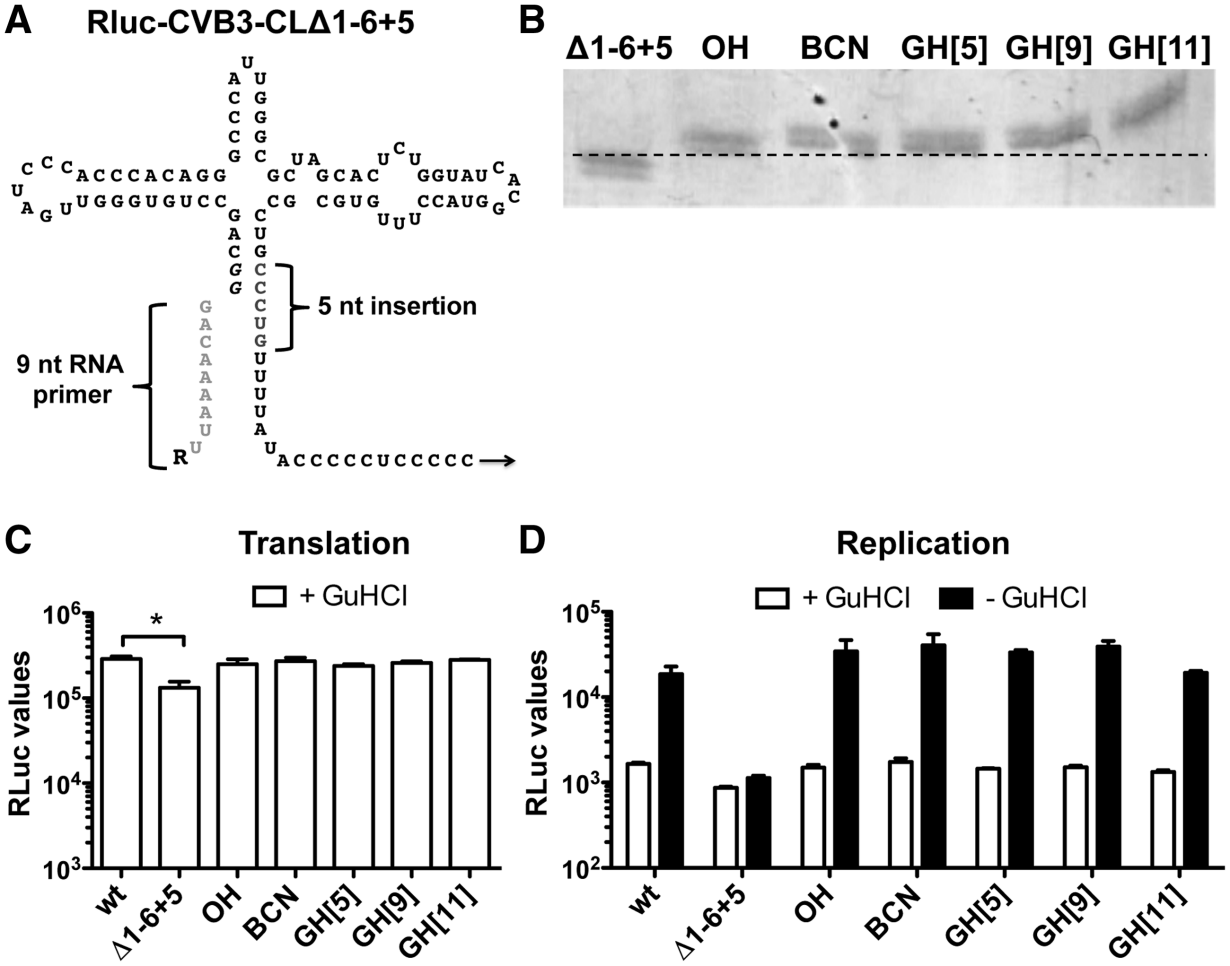
Base pairing-directed RNA primer ligation to RLuc-CVB3-CLΔ1−6 + 5 genomic RNA. (**A**) Schematic representation of the CLΔ1−6 + 5-modified CL structure. The 5-nt insertion in the 3′ strand of stem A is indicated in gray. Note that T7 RNA polymerase-transcribed RNA contains two additional guanine nucleotides (*italic*), which form base pairs with cytosine nucleotides of the 3′ strand of stem A. The 9-nt RNA primer used for base pairing-directed RNA ligation is depicted in light gray, and ‘R’ represents the different 5′ modifications. (**B**) RNA primer ligation efficiency to genomic RNA possessing the CLΔ1−6 + 5 structure was determined by urea–PAGE analysis of a 250-nt RNase H-digested 5′-terminal fragment. Note that ligation of the RNA primer reduces migration speed of the 250-nt RNase H-digested RNA fragment. (**C**) Translation of incoming genomic RLuc-CVB3 RNA (wt), RNA holding the mutated CL (Δ1−6 + 5) and RNA ligation products possessing different 5′ modifications (OH, BCN, GH[5], GH[9], GH[11]) were determined by transfection of RNA in HeLa cells in the presence of GuHCl. Eight hours post-transfection, HeLa cells were lysed and RLuc values were determined. Data from a representative experiment are presented as the mean of duplicate ±SD and analyzed using unpaired *t*-test (* indicates significant difference *P* < 0.05). (**D**) RNA replication of the transfected RNA was determined by comparing the RLuc values in the presence of GuHCl inhibitor (+ GuHCl) and in the absence of the inhibitor (− GuHCl). Data from a representative experiment are presented as the mean of duplicate ±SD. Note that the modification of the CL structure (Δ1−6 + 5) hampered RNA replication. However, reconstituting the stem by RNA primer ligation restored RNA replication again.

To allow the analysis of 5′ modifications of genomic RLuc-CVB3 RNA on both translation as well as replication, a new infectious clone was generated. This new clone holds the same deletion of 6 nt in the 5′ strand of stem A but now possesses only a 5-nt insertion in the 3′ strand of stem A (CLΔ1-6 + 5, Figure 2A). This new mutated CL structure allowed the ligation of 9-nt RNA primers (Figure 2A) devoid of any 5′ modification (OH) or a bicyclo[6.1.0]nonyne (BCN) group (26). Additionally, we also used three RNA primers holding a partial VPg peptide linked via the natural phosphodiester bond [GH(5), GH(9) and GH(11)] (28). Again the RNA primer ligation was very efficient (Figure 2B).

When the RNA ligation products were used for an RLuc translation assay in HeLa cells, mutation of the CL structure (CLΔ1-6 + 5) again showed a slight, but significant, effect on RLuc translation (Figure 2C). As observed before, repair of the CL structure by RNA primer ligation restored RLuc translation back to levels similar as wt (Figure 2C). Interestingly, in contrast to the CL + 8 modified stem (Figure 1F), the smaller extension of stem A did not affect replication of the genomic RNA (Figure 2D). Note that the RNA ligation products containing the partial VPg peptides [GH(5), GH(9) and GH(11)] are linked via the natural phosphodiester bond and therefore should be released by the TDP2 enzyme. On the contrary, the bond between the RNA and the BCN group is not a known substrate for TDP2. Nonetheless, this BCN-containing RNA was still efficiently translated and replicated with similar efficiency as wt RNA (Figure. 2C and D). Thus, small modifications, like a BCN group, at the 5′terminus of genomic RNA do not affect translation or replication.

### Coupling of VPg via a ‘non-cleavable’ bond to the genomic RNA via SPAAC and RNA ligation

**Figure 3.**
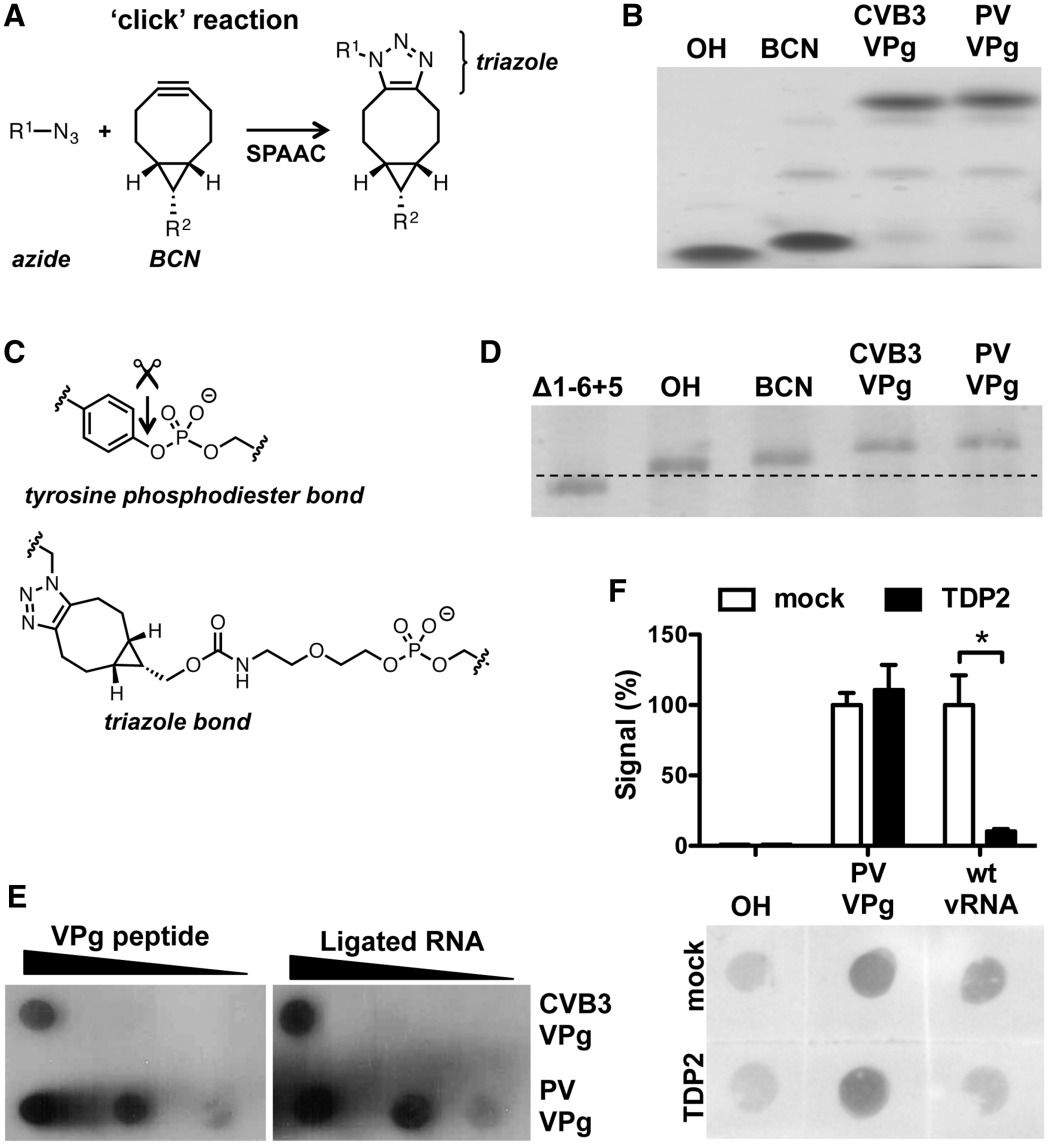
Modification of the 5′ terminus of picornavirus genomic RNA with VPg linked via a ‘non-cleavable’ bond. (**A**) Schematic representation of the SPAAC ‘click’ reaction that was used to couple the VPg peptides to the RNA primer (**B**) SPAAC ‘click’ reaction efficiency was determined by urea–PAGE analysis. The unmodified RNA primer (OH) and the BCN-modified RNA primer (BCN) migrated faster than the VPg-containing BCN primers (CVB3 VPg and PV VPg). (**C**) Structure of VPg-RNA linked either by the natural tyrosine phosphodiester bond or the triazole linkage. Arrow indicates the unlinkase site of the TDP2 enzyme. (**D**) RNA primer ligation efficiency to genomic RLuc-CVB3-Δ1-6 + 5 RNA was determined by urea–PAGE analysis of a 250-nt RNase H-digested 5′-terminal fragment. Note that ligation of the RNA primer reduces migration speed, especially in the case of the RNA primers containing VPg (CVB3 VPg and PV VPg). (**E**) The presence of VPg was determined by dot blot analysis. Equimolar amounts of the VPg peptide and RNA possessing VPg via a ‘non-cleavable’ bond were spotted on a membrane, and VPg presence was detected by a polyclonal antibody (29). Note that CVB3 VPg is less reactive than PV VPg as the antibody is raised against the PV VPg. Importantly, the signals of the peptides correlated with the signal intensities from the RNA ligation products possessing VPg. (**F**) Unlinkase reaction using recombinant TDP2 was performed using the RNA ligation product containing the unmodified RNA primer (OH), the PV VPg peptide (PV VPg) linked via a ‘non-cleavable’ bond and genomic RNA isolated from PV virions (wt vRNA). Values were corrected for background signal (OH), and mean of two independent experiments are shown ±SD and analyzed using unpaired *t*-test (* indicates significant difference *P* < 0.05). Note that TDP2 treatment reduced only the signal from viral RNA isolated from virions (wt vRNA) and not from RNA modified with PV VPg linked via a ‘non-cleavable’ bond (PV VPg).

BCN is a reactive cyclic alkyne that can be used to link azide-containing molecules via a SPAAC, also called copper-free ‘click’ reaction (Figure 3A) (26,34,35). To this end, we ordered azide-containing VPg peptides from both CVB3 and PV where the tyrosine at position 3 was replaced by a γ-azidohomoalanine residue. The BCN-containing RNA primer was efficiently coupled to the VPg peptides by SPAAC, resulting in a slower migrating RNA fragment following urea-PAGE analysis (Figure 3B). The resulting triazole bond is larger than the natural phosphodiester bond (Figure 3C) and should be ‘non-cleavable’ by the TDP2 enzyme. The RNA primers containing VPg linked via a ‘non-cleavable’ bond were successfully ligated to the CLΔ1-6 + 5 genomic RNA (Figure 3D), and the presence of VPg on the genomic RNA was further confirmed by dot blot analysis. Equimolar amounts of VPg-containing genomic RNA and VPg peptides were spotted on a membrane and a PV-VPg antibody (29) bound to these samples with a similar efficiency (Figure 3E), suggesting that the majority of RNA contained VPg. To confirm that the unnatural triazole bond between VPg and the genomic RNA is ‘non-cleavable’ by the TDP2 enzyme, RNA ligation product containing PV VPg and an equimolar amount of viral RNA isolated from PV virions, hence possessing VPg via a natural phosphodiester bond, was treated with the TDP2 enzyme (21,30). As shown in Figure 3F, only VPg linked via the natural phosphodiester bond (wt vRNA) was released from the genomic RNA, while the BCN-VPg bond (PV VPg) was unaffected. Taken together, we were able to attach VPg to genomic viral RNA via a SPAAC reaction, which is ‘non-cleavable’ by the TDP2 enzyme.

### The ‘non-cleavable’ bond does not interfere with protein translation or RNA replication

**Figure 4.**
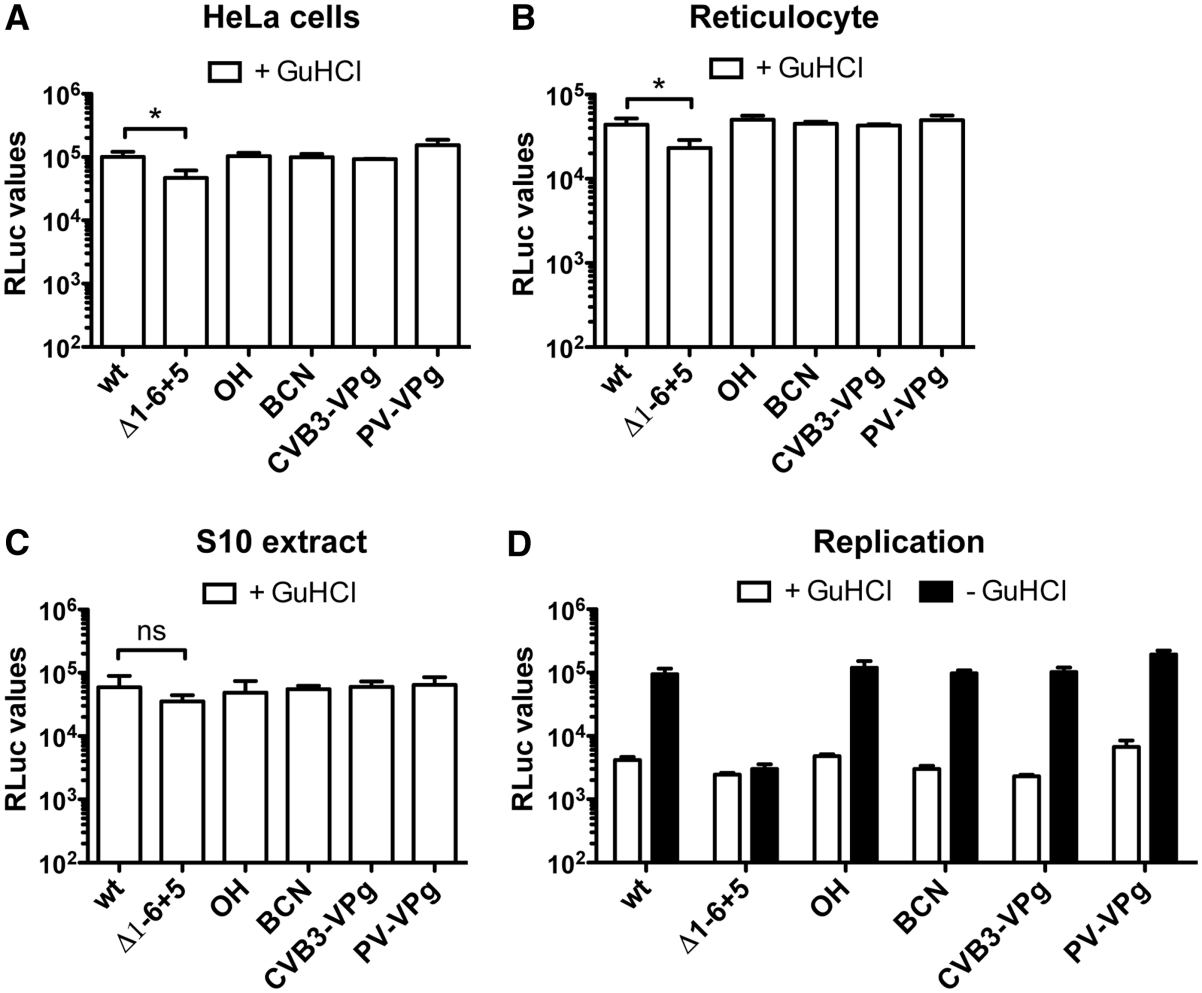
VPg unlinking is not required for picornavirus RNA translation and replication. (**A**) Translation of the incoming genomic RLuc-CVB3 RNA (wt), RNA holding the mutated CL (Δ1 − 6 + 5), RNA ligation products possessing a hydroxyl (OH) or a BCN group (BCN) at the 5′-end, and RNA holding VPg via a ‘non-cleavable’ bond (CVB3-VPg and PV-VPg) were determined by transfection of RNA in HeLa cells in the presence of GuHCl. Eight hours post-transfection, HeLa cells were lysed and RLuc values were determined. Data from a representative experiment are presented as the mean of triplicate ±SD and analyzed using unpaired *t*-test (* indicates significant difference *P* < 0.05). (**B**) Translation of the incoming genomic RLuc-CVB3 RNA (wt), RNA holding the mutated CL (Δ1−6 + 5) and RNA ligation products in RRL System (Promega) and (**C**) in HeLa S10 extracts. RLuc values were determined after 30 min of incubation. Data from a representative experiment are presented as the mean of triplicate ±SD and analyzed using unpaired *t*-test (* indicates significant difference *P* < 0.05, ns indicates no significant difference). As observed in HeLa cells, the 5′modification did not affect translation efficiency. (**D**) RNA replication of the transfected RNA was determined by comparing the amount of RLuc values in the presence of GuHCl inhibitor (+GuHCl) and in the absence of the inhibitor (−GuHCl). Data from a representative experiment are presented as the mean of triplicate ±SD. Note that the modification of CL structure (Δ1 − 6 + 5) hampered RNA replication. However, reconstituting the stem by RNA primer ligation restored RNA replication. Importantly, neither modification at the 5′ terminus affected RNA replication.

Using the genomic RNA possessing VPg via a ‘non-cleavable' bond, we were able to study the prerequisite of VPg unlinkase for translation of the incoming RNA and successive RNA replication. RNA ligation products were subjected to the RLuc translation assay in HeLa cells. As observed before, ligation of the BCN-modified RNA primer to the CLΔ1-6 + 5 genomic RNA resulted in similar translation levels as RNA lacking a 5′ modification (OH) and the wt RNA (Figure 4A). Importantly, also RNA modified with VPg via a ‘non-cleavable’ bond showed efficient RLuc translation. Therefore, it seems that, at least in this assay system, the inability to release VPg from the genomic RNA did not affect RNA translation. To confirm these results, alternative translation systems such as the HeLa S10 extract (31,32) and rabbit reticulocyte extracts (24,33,36) were also tested. In the rabbit reticulocyte extracts (Figure 4B) as well as HeLa S10 extracts (Figure 4C), the inability to release VPg from the genomic RLuc-RNA did not affect RLuc translation levels. Thus, VPg release from the genomic RNA is dispensable for translation of the incoming viral RNA.

To test whether the inability to release VPg interfered with RNA replication, an RNA replication assay was performed in the presence and absence of GuHCl. Also, in this replication assay, the ‘non-cleavable’ VPg bond did not negatively influence the RNA replication levels (Figure 4D). These data combined suggest that the release of VPg from the genomic RNA is not a prerequisite for RNA translation and replication.

## DISCUSSION

Over 35 years ago, the discovery was made that genomic RNA of picornaviruses possessed a covalently linked VPg peptide. Soon after this discovery, it was recognized that VPg is released from the incoming genomic RNA by a cellular enzyme. The importance of VPg unlinkase for efficient translation and/or replication has remained unknown ever since. It has been technically difficult to study the possible importance of VPg unlinkase because modification of the 5′ terminus of large RNA molecules, like the picornavirus genomic RNA, has proven to be challenging. Here we present a novel method to covalently derivatize the 5′ terminus of large RNA molecules with a diverse set of modifications. We exploited the highly folded CL structure in the 5′ UTR to allow base pairing-directed ligation of modified RNA primers. Combining this RNA ligation method with the new ‘click’ chemistry principle, we were able to modify the large genomic RNA with VPg via a ‘non-cleavable’ bond. Using these modified genomic RNA molecules in a series of translation and replication assays, we show that the inability to release VPg from the incoming viral RNA does not affect translation and replication efficiency.

For the ‘click’ reaction, we used a novel ring-strained alkyne, the BCN group (26). The main advantage of BCN over the other common cyclooctynes like DIBO, DIBAC or BARAC is the rather small size, the straightforward synthesis protocol and low lipophilicity (26). BCN reacted efficiently with the azide-modified VPg, and the resulting triazole-containing linkage between VPg and the genomic RNA, although somewhat longer, mimics the natural tyrosine phosphodiester structure (Figure 3C).

In contrast to the viral RNA isolated from virions, the ‘clicked’ VPg was not released from the RNA by the TDP2 enzyme in the *in vitro *unlinkase assay (Figure 3F). Most likely, the absence of the aromatic ring adjacent to the phosphodiester bond, which plays an important role in ligand recognition by TDP2 (37–39), results in the ‘non-cleavable’ linkage. However, as result of the low sensitivity of the dot blot analysis, we were unable to confirm the presence of VPg following transfection of cells with modified genomic RNA. Therefore, it cannot be ruled out that, for instance, cellular proteolytic activity might cleave the VPg peptide, but not at the triazole structure, which has been described to be extremely inert, as alkyne- and azide-containing molecules are not typically found in biological molecules (26,34,35). Recently, it has been shown that this triazole bond can only be reversed via strong mechanical force induced by, for instance, ultrasound (40). However, we performed our translation and replication assays under native conditions, and therefore the unnatural bond that is created between the genomic RNA and VPg via the ‘click’ reaction will most likely be retained after transfection of the RNA.

The presence of VPg linked via a ‘non-cleavable’ bond at the 5′ terminus of the genomic RNA did not affect translation of the transfected RNA (Figure 4A–C). A previous study already showed that VPg-containing genomic RNA was able to associate with ribosomes (24). However, because these authors performed their assays under conditions that impaired translation, they were unable to determine whether VPg-containing RNA could be translated. Our study extends these data and shows that the presence of VPg at the 5′ terminus of the genomic RNA does not impair translation as well as replication. This important new piece of data consequently argues against some of the original speculation that the presence of VPg might prevent efficient translation of viral RNA, as discussed in (15,16,23). However, it should be noted that the translation data presented in our study were obtained after RNA transfection or in cell-free extracts. It is possible that viral translation requirements are different when picornavirus virion RNA is delivered to the cell cytoplasm following uncoating of entering virus particles.

If the presence of VPg is not affecting translation or replication of the incoming viral RNA, why is this peptide released from the genomic RNA on introduction in the cytoplasm? It has been suggested that VPg might play a role in encapsidation of the genomic RNA in particles (16,20,21), as virions only contain VPg-containing viral RNA (15,41). Unlinkase of VPg from the viral RNA could mark the RNA exclusively for translation and replication. This suggestion is in line with the recent observation that the TDP2 enzyme is relocated to cytoplasmic sites distal to the viral RNA late in infection (21), which correlates with the shift from RNA translation/replication to RNA encapsidation. Thus, VPg release may mark viral RNA for translation and replication, but this study clearly shows that it is not required for these processes.

## FUNDING

The Netherlands Organization for Scientific Research [NWO-825.11.022 to M.A.L. and NWO-017.006.043 to Q.F.]; the National Institutes of Health [AI026765 to B.L.S.]; and a Graduate Research Fellowship from the U.S. National Science Foundation (to S.M.). Funding for open access charge: the Netherlands Organization for Scientific Research (NWO).

*Conflict of interest statement*. None declared.
